# Susceptibility of diverse sand fly species to Toscana virus

**DOI:** 10.1371/journal.pntd.0013031

**Published:** 2025-05-02

**Authors:** Magdalena Jancarova, Nikola Polanska, Adrien Thiesson, Frédérick Arnaud, Marketa Stejskalova, Marketa Rehbergerova, Alain Kohl, Barbara Viginier, Petr Volf, Maxime Ratinier

**Affiliations:** 1 Laboratory of Vector Biology, Department of Parasitology, Faculty of Science, Charles University, Prague, Czech Republic; 2 IVPC UMR754, INRAE, Universite Claude Bernard Lyon 1, EPHE, Université PSL, Lyon, France; 3 MRC-University of Glasgow Centre for Virus Research, Glasgow, United Kingdom; 4 Centre for Neglected Tropical Diseases, Departments of Tropical Disease Biology and Vector Biology, Liverpool School of Tropical Medicine, Liverpool, United Kingdom; Medizinische Universitat Wien, AUSTRIA

## Abstract

Toscana virus (TOSV) is an emerging but neglected human pathogen currently circulating around the Mediterranean basin including North Africa. Human illness ranges from asymptomatic or mild flu-like syndromes to severe neurological diseases such as meningitis or meningoencephalitis. Despite its significant impact, understanding of TOSV transmission and epidemiology remains limited. Sand flies (Diptera: Phlebotominae), specifically *Phlebotomus perniciosus* and *Phlebotomus perfiliewi*, are believed to be the primary vectors of TOSV. However, the spread of TOSV to new geographical areas and its detection in other sand fly species suggest that additional species play a role in the circulation and transmission of this virus. This study investigated the vector competence of four sand fly species - *P. tobbi*, *P. sergenti*, *P. papatasi*, and *Sergentomyia schwetzi* - for two TOSV strains: 1500590 (TOSV A lineage) and MRS20104319501 (TOSV B lineage). Sand flies were orally challenged with TOSV via bloodmeals. None of the tested species showed susceptibility to the TOSV A strain. However, for TOSV B strain, *P. tobbi* demonstrated a high potential as a new vector, exhibiting high infection and dissemination rates. *P. sergenti* also showed some susceptibility to TOSV B, with the virus dissemination observed in all infected females. These finding suggests that *P. tobbi* and *P. sergenti* are new potential vectors for TOSV B. Given that *P. tobbi* and *P. sergenti* are the primary vectors of human leishmaniases in the Balkans, Turkey and Middle East, their susceptibility to TOSV could have significant epidemiological consequences. On the other hand, *P. papatasi* and *S. schwetzi* appeared refractory to TOSV B infection. Refractoriness of *P. papatasi,* a highly anthropophilic species distributed from the Mediterranean to the Middle East and India, suggests that this species does not contribute to TOSV circulation.

## Summary

Sand flies are blood-sucking insects that transmit various human pathogens, including parasitic protists of the genus *Leishmania* and several viruses, such as the phlebovirus Toscana (TOSV). TOSV infections typically result in flu-like symptoms, but in some patients, the infection can affect the central nervous system (CNS) or even be fatal. Due to climatic and environmental changes associated with human activity, it is likely that sand flies and the pathogens they transmit will spread beyond current boundaries. However, the specific sand fly species that transmit TOSV are not well understood. Therefore, it is essential to identify vectors that are involved in the circulation and transmission of the virus. In our study, we found that the range of potential TOSV vectors is broader than previously thought and that individual virus strains may influence vector competence. Specifically, we demonstrated that *Phlebotomus tobbi* and *P. sergenti,* two sand flies common in Mediterranean area and Middle East, are susceptible to TOSV and likely transmit this virus in nature.

## Introduction

*Phlebovirus toscanaense* (Toscana virus, TOSV) is an emerging yet neglected human pathogen (genus *Phlebovirus*, family *Phenuiviridae*, order *Haeravirales* [1]) that is transmitted by sand flies. Symptoms in humans vary from non-symptomatic to febrile illness to (occasionally fatal) central nervous system diseases. TOSV is considered to be one of the three most important causes of aseptic meningitis in the countries where this virus circulates [2]. TOSV has been reported in many countries around the Mediterranean basin, including Southern Europe (Italy, Spain, France, Portugal), South-Eastern Europe (Turkey, Cyprus), the Balkan peninsula (Greece, Croatia, Bosnia and Herzegovina, Kosovo, Bulgaria), North Africa (Morocco, Algeria, Tunisia) and Mediterranean islands (reviewed by [3,4]). Popescu et al. [5] described the occurrence of neuroinvasive TOSV infections in Romania.

TOSV is currently divided into three genetic lineages: A, B and C [3], however, the latter was only characterised based on partial sequences, and whole genome sequences or virus isolates have never been obtained [6]. Thus far, no biological differences between these different genetic lineages have been described with regards to host infection, clinical signs, or disease severity [2,5] though their geographical distribution may differ (reviewed by [7]). Despite the wide geographical distribution of this pathogen and the high incidence of summer meningitis and encephalitis around the Mediterranean basin [8], data on TOSV biology and epidemiology are very limited.

To date, vertebrates infected by TOSV appear to be dead-end hosts that do not play a crucial role in TOSV circulation [3]. Viremia in humans is low and transient (24–36 hours) [9] and dogs do not develop significant viremia after experimental infection [10]. Anti-TOSV antibodies or/and viral RNA were found in humans [11–13], livestock, horses, dogs and cats [14–18], bats [19] as well as birds [20,21]. However, there are only two studies describing isolation of the virus from non-human hosts; the bat *Pipistrellus kuhli* (brain) [22] and birds (pigeons, mallards, partridges) [23]. Overall, the life cycle of TOSV in nature remains unclear.

It is generally accepted that TOSV is transmitted by phlebotomine sand flies [3,24–27]. Sand flies (Diptera: *Psychodidae*) are hematophagous insects known as vectors of human and animal pathogens, such as protozoan parasites (namely *Leishmania* sp.), bacteria (e.g., *Bartonella bacilliformis*) and viruses [28,29]. Both male and female sand flies feed on natural sugar sources, such as plant sap or honeydew, but females also feed on vertebrate blood to obtain proteins and nutrients necessary for egg development [28,29]. Until now, only two sand fly species, *Phlebotomus perniciosus* and *P. perfiliewi* are considered as vectors of TOSV. However, the seroprevalence of TOSV in humans and animals in Eastern Mediterranean countries, where *P. perniciosus* is not present and *P. perfiliewi* is rare, suggests the involvement of other sand fly species in the circulation of the virus [3]. Interestingly, TOSV was detected in *Sergentomyia minuta* in Marseille [30], *P. tobbi* in Cyprus [31], *P. neglectus* in Croatia [16], *P. longicuspis* and *P. sergenti* in Morocco [32–34] and in *P. major*, *P. papatasi* and *S. dentata* in Turkey [35]. However, the role of these sand fly species in TOSV transmission remains to be elucidated as virus detection does not mean necessary transmission.

The two main objectives of this study were to identify novel TOSV vectors and to determine whether distinct TOSV strains differ in their interactions with the insect vector. This information is crucial not only to fill scientific knowledge gaps but also to better understand the ecology and epidemiology of TOSV. Moreover, due to climate change, animal migration and human activities, sand flies expanded to new areas along with the pathogens that they transmit [36]. Therefore, this knowledge is essential to assess the risk of TOSV expansion and implement adequate transmission control strategies.

## Materials and methods

### Sand flies

Four sand fly species were used for experimental infections: *P. tobbi*, *P. sergenti*, *P. papatasi* (all three colonies originating from Turkey) and *S. schwetzi* (colony originating from Ethiopia). All colonies have been established for many generations at the Laboratory of Vector Biology at Charles University, Prague and tested for the presence of phleboviruses and *Wolbachia* sp.. All colonies’ tests were negative. More detailed information on the establishment and maintenance of sand fly colonies was published elsewhere [37]. Three-to-seven-day-old females were used in all experiments and were maintained at 26°C with 50% sucrose after infection.

### Cell culture

All experiments were done with either VeroE6 cells (obtained from Philippe Marianneau, Unité de virologie - ANSES Lyon, France) or BSR cells (BHK21 clone, kindly provided by K. K. Conzelmann) [38]. Cells were cultivated in Dulbecco’s Modified Eagle medium with high glucose, L-glutamine, sodium pyruvate and phenol-red (DMEM) supplemented with 10% foetal bovine serum (FBS) and 1% penicillin-streptomycin (P/S), in 75 cm^2^ plastic tissue culture flasks with filtered screw cap, placed horizontally in a CO_2_ incubator (5% CO_2_, 37°C).

### Virus

Experimental infections of sand flies were performed with TOSV strain 1500590 (Lineage A, named TOSV A: kindly provided by Philippe Marianneau, GenBank Accession No: MT032306, MT032307 and MT032308) and strain MRS2010–4319501 (Lineage B, called TOSV B; purchased at European Virus Archive collection, GenBank Accession No: KC776214, KC776215 and KC776216) [39,40]. TOSV stocks were produced by infecting BSR cells, cultivated in DMEM supplemented with 4% FBS, with either TOSV A or TOSV B. Supernatants were collected when cytopathic effect (CPE) was evident (usually 4 days post-infection, d.p.i.) and clarified by centrifugation at 500g for 5 min at 4°C. Viral suspensions were subsequently deposited at the top of 20% sucrose cushion (w/v, diluted in PBS) and ultracentrifugated at 124,000g for 3 hours at 4°C. Pellets were resuspended in PBS and stored at -80°C. Viral stock titers were determined by plaque-forming assays using veroE6 cells. Briefly, virus stocks were diluted in a 10-fold manner and incubated on VeroE6 cells for 2 hours at 37°C with 5% CO_2_. Cells were washed twice with PBS and overlayed with a volume-to-volume mixture of 2.5% ultrapure agarose solution (w/v) and 2X minimal essential medium (MEM 2X), supplemented with 4% FBS. Cells were incubated at 37°C, 5% CO_2_ for 6 days to allow viral plaques to grow to visible sizes, before being fixed using 4% formaldehyde solution and stained using crystal violet solution. Viral plaques were then counted, and viral titers are expressed as PFU/ml (plaque-forming unit per ml).

### Sand fly infections

All infectious experiments were performed in the BSL2 laboratory. In each experiment, three groups of 120 sand fly females were used: a first group for blood feeding without the virus as control group, a second group for infection with TOSV lineage A and a third group for infection with TOSV B. All experimental infections were carried out in a glove box; firstly, female sand flies from the control group were allowed to feed through the chick skin membrane on heat-inactivated ram blood (LabMediaServis s.r.o.) for 90–120 min (more detailed information about feeding system was published elsewhere [37]). Subsequently, groups for infectious bloodmeal were fed one by one through the chick skin membrane on heat-inactivated ram blood containing an infectious dose of TOSV A or TOSV B (approx. 10^6^ PFU/ml) of blood in the same mode as for control group without virus. Subsequently, non-fed sand fly females were removed by a battery-powered aspirator. All experiments were done within 3 years. The results were consistent.

### Sand fly dissections and sample processing

After blood feeding, two blood fed females from each group were homogenized individually by a crushing pestle in 1 ml of crushing medium (DMEM, 4% FBS, amphotericin B 2.5 g/ml, nystatin 100 U/ml, gentamycin 50 g/ml, penicillin-streptomycin 50 g/ml), aliquoted into two samples (each 500 µl) and stored at -80°C as a control of the feeding process (at day 0 post infection - D0 p.i.), to see if the virus stayed infectious during the feeding process. All sand fly species tested were kept at 26°C and humidity above 70%. Short-living females of *P. tobbi* were dissected on days 4 and 8 post infection (D4, D8 p.i.), while females of *P. sergenti*, *P. papatasi*, and *S. schwetzi* were also dissected on day 14 (D14 p.i.). Additionally, due to the high survival rate of *S. schwetzi*, an extra dissection was performed on day 18 post infection (D18 p.i.). Sand fly females were anesthetized on ice and they were dissected in the glove box under a binocular microscope, in a drop of PBS to head with attached salivary glands (H) and rest of the body (B) to determine TOSV presence. These body parts were homogenized as described above, aliquoted to 500 µl) and stored at -80°C. Thus, for each sample - the body of one sand fly female or the head of one sand fly female, we had two aliquots (the first aliquot for TCID_50_ and the second for quantitative PCR - qPCR, see below), each with a volume of 500 µl. Every sample has a unique code for species – letter (“T” – *P. tobbi*, “E” – *P. sergenti*, “P” – *P. papatasi*, “C” – *S. schwetzi*) and sample numbers are listed in S1 Table.

### Virus detection by end point dilution assay (TCID_50_)

Ninety-six well plates were filled with 100 µl DMEM medium supplemented with 4% FBS and 1% P/S except for the first row, where 111 µl of each sample were added. All samples were tested in quadruplicates and for each series of experiments females fed with non-infected blood were also used as a negative control. Serial dilution of the sample was done by transferring 11 µl from one line to the other until the last line where 11 µl was removed and discarded. Subsequently, 100 µl of VeroE6 cells (concentration at 4x10^4^ cells/ml) were added to each well. Plates were incubated for 5 days in a CO_2_ incubator (5% CO_2_, 37°C) and then the presence of CPE caused by the virus was assessed under an inverted microscope. Wells showing CPE were scored as positives when wells with no apparent CPE were considered negatives. In each endpoint dilution assay, two types of controls were included: wells containing only cells and wells with homogenate from blood-fed females in the control group. This allowed for a clear detection of the CPE of TOSV on the cell monolayer. Viral titers were calculated by using Reed and Muench’s method and expressed as tissue culture infectious dose 50 per millilitre (TCID_50_/ml) [41].

### Isolation of TOSV RNA and cDNA synthesis from experimental samples

Detection of TOSV RNA by qPCR in the experimental samples was carried out to determine viral RNA levels. This method was used to support the data from TCID_50_ and all the positive samples and samples on the detection limit of the TCID_50_ assay were tested, as well as some randomly chosen negative samples from TCID_50_. From these analysed samples, TOSV RNA was isolated from a 500 µl aliquot using QiaAmp Viral RNA Mini Kit (Qiagen). The 500 µl was divided by half and 1000 µl AVL buffer with acyl carrier RNA added to each aliquot and incubated for 10 min at room temperature. RNA isolation steps were carried out according to the manufacturer’s protocol and the RNA was eluted in two steps each with 40 µl AVE buffer (1 min, 8000 rpm). Two protocols were used for cDNA synthesis - one to produce positive strands and one for negative strands. cDNA synthesis was performed by using QuantiTect Reverse Transcription kit (Qiagen) by using 12 µl of RNA samples. cDNAs specific to the TOSV negative strand of the S segment were obtained with primer STOS-50F primer, cDNA specific to the TOSV positive strand of the S segment were synthesised using primer STOS-138R [42]. cDNA samples were stored at -20°C until used.

### Preparation of the TOSV RNA standard curve for quantitative PCR

For RNA standard production, DNA templates were produced by PCR on plasmids containing either TOSV A or TOSV B antigenome of S segment using CloneAmp Hifi PCR Premix (Takara) and qPCR_S_TOSV_RT (ACACAGAGATTCCCGTG) and qPCR_S_TOSV_T7_rev (TAATACGACTCACTATAGGGCCATGAGCATCAGCAATRGTGG) primers. To enable the production of the genomic viral RNA, the T7 RNA polymerase promoter was added at the 5’ termini of qPCR_S_TOSV_T7_rev primer (underline sequence). Briefly, the PCR mixture (final volume 25 µl) contained 12.5 µl of Clone Amp Premix, 1 µl forward primer (10 µ M), 1 µl reverse primer (10 µ M), 8.5 µl of PCR-grade H_2_O, and 2 µl of plasmid (5 ng/ µ L). PCR, 30 cycles: 10 sec 98°C, 10 sec 55°C, and 5 sec 72°C. PCR products (25 µl) were then digested with 0.5 µl Dpnl enzyme (NEB) for 1 hour at 37°C. Subsequently, digested products were loaded on horizontal gel electrophoresis on 1.5% agarose gel (35 min, 100 V) and then visualised. The digested product was cut from the gel and cleaned up by the NucleoSpin Gel and PCR Clean-up kit (Macherey-Nagel) and DNA concentration was measured on NanoDrop One (ThermoFisher Scientific).

The purified DNA template was used to produce single-stranded negative sense TOSV S-segment RNA with the T7 RiboMAX (Promega) production system. Briefly, the reaction of 20 µl contained: 10 µl of RiboMAX Express T7 2X Buffer, 2 µl of Enzyme Mix, T7 Express, 125 ng of linear DNA template, and filled up to 20 µl by PCR-grade H_2_O. RNA was produced in a PCR thermocycler at 37°C for 30 min and then cooled down. RNA was then purified using the QIAamp Viral RNA Mini kit (Qiagen). The purified RNA was cleaned from template DNA contaminations by using the TURBO DNA-free Kit (Invitrogen). Before DNase treatment, RNA was incubated for 10 min at 65°C followed by 5 min on ice to increase DNase efficiency. DNase treatment was followed according to the manufacturer’s protocol. Briefly, 50 µl reaction contains 5 µl of 10X TURBO DNase Buffer, 30 µl of RNA produced with T7 RiboMAX, and 14 µl by PCR-grade H_2_O. This mixture was incubated for 30 min at 37°C, and then 0.5 µl of TURBO DNase Enzyme (2 Units/μl) were added to the reaction mixture, which was subsequently incubated for another 30 min at 37°C. After this, DNase inactivation buffer was added to the reaction mixture for 5 minutes at room temperature and the mix was centrifugated for 2 minutes at 10,000 g. The supernatant was collected to obtain pure RNA.

The concentration and purity of RNA were checked on NanoDrop One (A260/A280) and the number of RNA copies was calculated according to the formula:

$RNA\;copy\;number/\mathrm{\backslash muL}\;=\;\frac{[RNA]g/\mathrm{\backslash muL}}{N\;\times 340}\times {6.02\times 10}^{23}$

N = RNA fragment length in bp

340 = Molecular weight of 1 bp

$6.02{\times 10}^{23}\;=\;Avogadro\;number$

Finally, the RNA was aliquoted in the working solutions with a concentration of 10^8^ RNA copies/ µl which were stored at -80°C until used and diluted as the standard curve for qPCR.

### Virus detection by quantitative PCR

Quantitative PCR was performed on the LightCycler 480 Real-Time PCR System (Roche) using the QuantiTect Probe PCR kit (Qiagen) in a total volume of the reaction of 10 µl in the LightCycler 480 Multiwell Plate 384 white (Roche). Each reaction contained 5 µl qPCR master mix, 0.4 µl forward primer (10 µ M), 0.4 µl reverse primer (10 µ M), 0.1 µl probe, 2.1 µl PCR-grade H_2_O, and 2 µl cDNA template (obtained by the transcription either positive or negative strand of S segment of TOSV RNA). Gene specific primers and probes for the TOSV S-segment were used [42]. The qPCR conditions were as follows: enzyme activation 15 min at 95°C and 45 cycles of denaturation (94°C, 15 sec) and annealing with extension step (60°C, 1 min), followed by a cooling step (40°C, 10 min). The results were analysed in LightCycler 480 SW 1.5.1 using “absolute quantification 2^nd^ derivative maximum for all samples” analyses. The samples and the calibration curve were measured every time in triplicate, the obtained Cp (crossing point) was averaged, and the standard deviation was calculated. If one Cp of the reactions did not correspond with the other two from triplicate, this reaction was manually excluded from the analysis and only a duplicate was used for the calculation of the final Cp of the sample. Subsequently, the genome copy number (RNA copies/µl) was counted from obtained Cp values and the standard curve for every sample. All TCID_50_ positive samples (both heads and bodies of one sand fly female), along with those on detection limit of TCID_50_ (lower than 1.78x10^2^ TCID_50_/ml, “Q” in S1 Table), were tested by qPCR. For some samples both 500 µl aliquots (one for TCID_50_ and the second for qPCR) were used for retesting of viral titer by TCID_50_, thus there was nothing left for qPCR (S1 Table).

### Statistical analysis and data visualisation

Samples above the TCID_50_ quantification limit of the TCID_50_ assays, and samples on or below the TCID_50_ quantification limit (at least one well positive in the first row) with confirmed presence of TOSV RNA by qPCR, were considered TOSV positive. For data representation, samples below quantification limit were arbitrarily assigned a titer value corresponding to half of the value of the quantification limit (*i.e.,* 8.9x10^1^ TCID_50_/ml). Data visualisation and statistical analysis were performed using R and RStudio softwares [43] (version 4.3.3 and 2023.12.1 + 402 respectively) and ggplot2 (https://CRAN.R-project.org/package=ggplot2) and rstatix packages (https://CRAN.R-project.org/package=rstatix). Pairwise comparisons of proportions tests were used as statistical analysis to compare infection and dissemination rates between TOSV A and TOSV B blood fed sand flies.

## Results

### Infection of *P. tobbi* by TOSV

**Fig 1:**
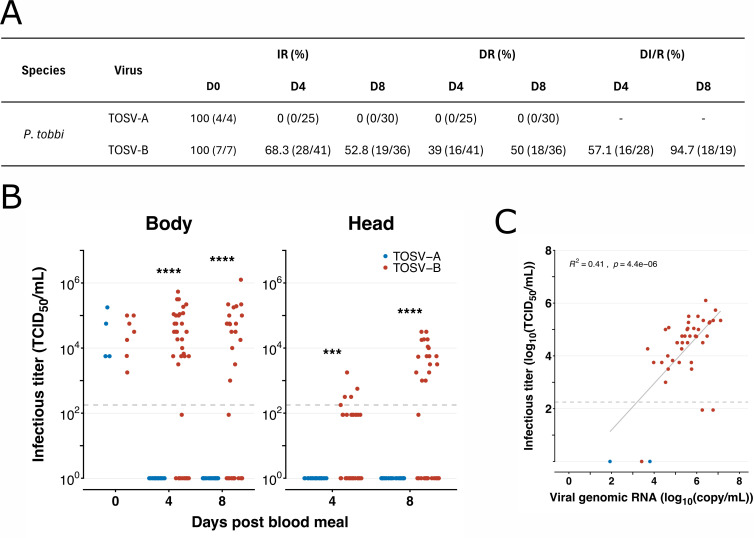
Susceptibility of *P. tobbi* to TOSV infection. *P. tobbi* sand flies were orally exposed to bloodmeal with either TOSV A or TOSV B (10^6^ PFU/ml) and collected at D4 and D8 p.i. to assess the presence of TOSV in bodies or heads with salivary glands. (**A**) Summary values of infection rate (IR), dissemination rate (DR) and dissemination rate among infected sand flies (DI/R). (**B**) Infectious viral titers were obtained by TCID_50_ limiting-dilution assays in body and head homogenates. Each dot represents a single sand fly. Light grey dashed lines indicate the threshold of virus detection (1.78x10^2^ TCID_50_/ml). Statistical significance between infection and dissemination rate was determined using pairwise comparisons of proportions tests: p < 0.001 (***) and p < 0.0001 (****). (**C**) Viral genome copy numbers were determined by qPCR and plotted against infectious viral titers. Light grey dashed lines indicate the threshold of virus quantification by TCID_50_ dilution assay (1.78x10^2^ TCID_50_/ml).

We first assessed the vector competence of *P. tobbi* for TOSV A and TOSV B strains through blood feeding. This experiment was repeated five times and data were pooled as this species does not feed very readily on an artificial feeding system under laboratory conditions. Together 55 and 77 females were dissected and tested for infection and dissemination of TOSV A and TOSV B, respectively. In total, 25 and 30 females were tested at D4 and D8 p.i. for TOSV A infection and dissemination (Fig 1A). None of these females were positive except at D0 p.i. where the mean value of viral titers was determined at 6.13x10^4^ TCID_50_/ml (Fig 1B); proving that blood contained infectious TOSV A. In the case of TOSV B, at D4 p.i., 28 out of 41 females tested positive for TOSV by TCID_50_ assay (infection rate of 68.3%) and 16 also showed a disseminated infection (57.1%) (Fig 1A). At D8 p.i., 19 out of 36 females were positive for TOSV B (infection rate of 52.8%) and 18 females exhibited a disseminated infection (94.7%) (Fig 1A). In the analyzed bodies, infectious viral titers at D4 p.i. and D8 p.i. ranged from 3.16x10^3^ to 5.41x10^5^ TCID_50_/ml and 1.00x10^3^ to 1.26x10^6^ TCID_50_/ml, respectively (Fig 1B). For the head fraction, viral titers ranged between 1.78x10^2^ and 1.78x10^3^ TCID_50_/ml at D4 p.i., and 1.00x10^3^ and 3.16x10^4^ TCID_50_/ml at D8 p.i. (Fig 1B).

Detection of TOSV negative-strand RNA by qPCR was used to confirm the results above. A total of 39 females (6 TOSV A, 33 TOSV B) at D4 p.i., and 20 females (5 TOSV A, 15 TOSV B) were tested at D8 p.i. The negative strand RNA of TOSV B was detected in 29 and 12 females at D4 and D8 p.i., respectively. Interestingly, TOSV A RNA was present in 2 females (6.3x10^3^ and 8.5x10^1^ RNA copies/µl) at D4 p.i. while none was detected at D8 p.i.. (Fig 1C).

To further confirm TOSV replication in sand flies, molecular detection of positive-strand viral RNA was also performed, as antigenome is produced during virus replication. Among TOSV-positive sand flies, 40 females were tested for TOSV-positive strand RNA (28 at D4 p.i. and 12 at D8 p.i.). At D4 p.i., 20 females were positive (74.1%), while 11 were at D8 p.i. (91.7%), demonstrating that TOSV is indeed replicating in infected females. Detailed information about the viral titers, Cp values and genome copy number, and the presence of positive strands of RNA are shown in S1 Table.

Taken together, these data confirm that TOSV A does not infect *P. tobbi*, whereas TOSV B is capable of infection, replication and dissemination in this species. This information is consistent between both TCID_50_ and molecular methods.

### Infection of *P. sergenti* by TOSV

**Fig 2:**
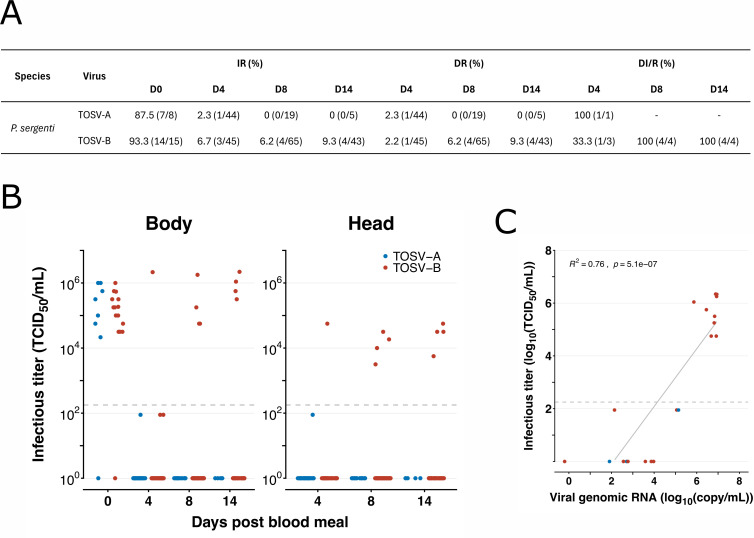
Susceptibility of *P. sergenti* to TOSV infection. *P. sergenti* sand flies were orally exposed to bloodmeal with either TOSV A or TOSV B (10^6^ PFU/ml) and collected at D4, D8 and D14 p.i. blood meal to analyse bodies and heads with salivary glands for the presence of TOSV. (**A**) Summary values of infection rate (IR), dissemination rate (DR) and dissemination rate among infected sand flies (DI/R). (**B**) Infectious viral titers were obtained by TCID_50_ limiting-dilution assays in body and head homogenates. Each dot represents a single sand fly. Light grey dashed lines indicate the threshold of virus detection (1.78x10^2^ TCID_50_/ml). (**C**) Viral genome copy numbers were determined by qPCR and plotted against Infectious viral titers. Light grey dashed lines indicate the threshold of virus quantification by TCID_50_ dilution assay (1.78x10^2^ TCID_50_/ml).

The experiment with *P. sergenti* was repeated ten times and data were pooled. Here, 68 and 153 succesfully blood fed females were dissected and tested for infection and dissemination with TOSV A and TOSV B, respectively. Out of all tested females, only 1 was positive for TOSV A at D4 p.i. albeit with disseminated infection (Fig 2A). Other positive females were only detected at D0 p.i., with a mean viral titer of 4.37x10^5^ TCID_50_/ml (Fig 2B). For TOSV B 45, 65 and 43 *P. sergenti* females were tested at D4, D8 and D14 p.i., respectively. At D4 p.i., 3 out of 45 females were positive (Fig 2A); 1 female defecated without visible blood remnants in the midgut and viral loads were measured at 2.15x10^6^ TCID_50_/ml in the body and 5.62 x10^4^ TCID_50_/ml in the head (Fig 2B), indicating dissemination of TOSV B. The 2 remaining females still had visible blood residues and detection of infection was at the limit of the TCID_50_ method, but it was confirmed by qPCR, without disseminated infection. At D8 and D14 p.i., 4 out of 65 and 4 out of 43 females were positive by the TCID_50_ assay, respectively, and showed disseminated infection (Fig 2A). For the bodies, the viral loads ranged from 5.62x10^4^ to 1.78x10^6^ TCID_50_/ml at D8 p.i. while, at D14 p.i., they were even higher – 3.16x10^5^ to 2.20x10^6^ TCID_50_/ml (Fig 2B). The viral loads in heads at D8 and D14 p.i. ranged from 3.16x10^3^ to 3.16x10^4^ TCID_50_/ml and 5.62x10^3^ to 5.62x10^4^ TCID_50_/ml, respectively (Fig 2B).

Subsequently, a total of 15 females (4 TOSV A, 9 TOSV B) at D4 p.i., 15 females (4 TOSV A, 13 TOSV B) at D8 p.i., and 14 females (3 TOSV A, 11 TOSV B) at D14 p.i. were tested by qPCR for the presence of negative strand TOSV RNA. The negative strand RNA of TOSV B was detected in 4, 7, and 6 females at D4, D8 and D14 p.i., respectively. In comparison, negative strands of TOSV A RNA were detected in 2 females at both D4 and D8 p.i. (negative by TCID_50_). Like *P. tobbi*, data from qPCR correlate with viral titers (Fig 2C).

All TOSV-positive females were tested for positive-strand viral RNA (4 at D4 p.i., 4 at D8 p.i. and 4 at D14 p.i.). TOSV antigenome was then detected in 3, 1 and 4 females at D4, D8 and D14 p.i. respectively (75%, 25% and 100%). All results are summarised in S1 Table.

This suggest that *P. sergenti* seems to be susceptible to TOSV B infection when both TCID50 and molecular assays confirmed virus infection, replication and dissemination. Its role in the circulation of TOSV A needs to be further clarified in the future.

### Infection of *P. papatasi* by TOSV

**Fig 3:**
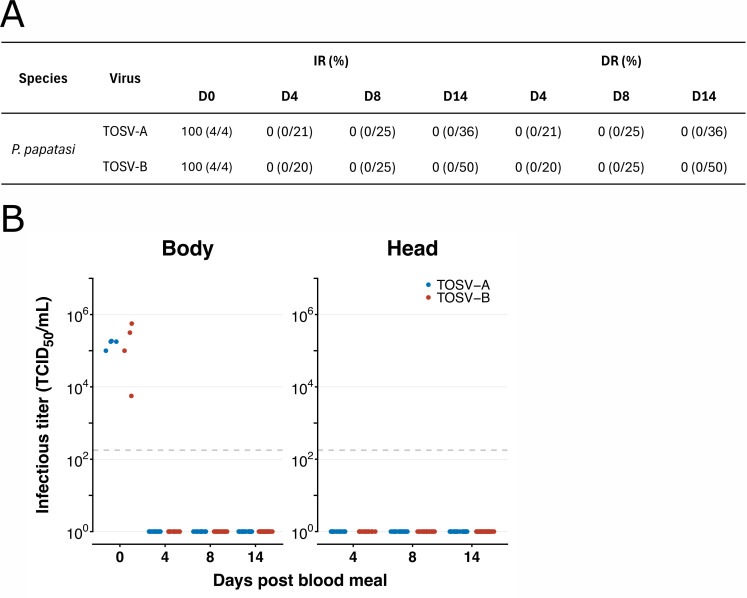
Susceptibility of *P. papatasi* to TOSV infection. *P. papatasi* sand flies were orally exposed to blood meal with either TOSV A or TOSV B (10^6^ PFU/ml) and collected at D4, D8 and D14 p.i. to analyse body and head with salivary glands for TOSV. (**A**) Summary values of infection rate (IR), dissemination rate (DR) among infected sand flies. (**B**) Infectious viral titers were obtained by TCID_50_ limiting-dilution assays in body and head homogenates. Each dot represents a single sand fly. Light grey dashed lines indicate the threshold of virus quantification (1.78x10^2^ TCID_50_/ml).

In two independent experiments, 82 (21 at D4 p.i., 25 at D8 p.i., 36 at D14 p.i.) and 95 (20 at D4 p.i., 25 at D8 p.i., 50 at D14 p.i.) *P. papatasi* females were assessed following infection assay with TOSV A and TOSV B, respectively (Fig 3A). All dissected and tested females were negative for both TOSV A and TOSV B except at D0 p.i. (demonstrating that blood meals contained infectious viral particles), with a mean viral titer of 2.46x10^5^ TCID_50_/ml for TOSV B and 1.60x10^5^ TCID_50_/ml for TOSV A (Fig 3B).

As performed previously, randomly selected samples were tested for the presence of negative strand TOSV RNA using qPCR. 27 females were tested: 9 at D4 p.i. (3 females for TOSV A, 6 females for TOSV B), 9 at D8 p.i. (3 females for TOSV A, 6 females for TOSV B), and 9 at D14 p.i. (3 females for TOSV A, 6 females for TOSV B). Only 1 female had a positive body for TOSV A RNA at D4 p.i., whereas RNA of TOSV B was detected in the bodies of 2 females at D14 p.i. These 3 samples were also positive for TOSV positive-strand RNA, however all measured Cp values were high (from 38.1 to 39.3). Results are summarized in S1 Table. Importantly, these results suggest that neither TOSV A and TOSV B can disseminate in *P. papatasi*.

### Infection of *S. schwetzi* by TOSV

**Fig 4:**
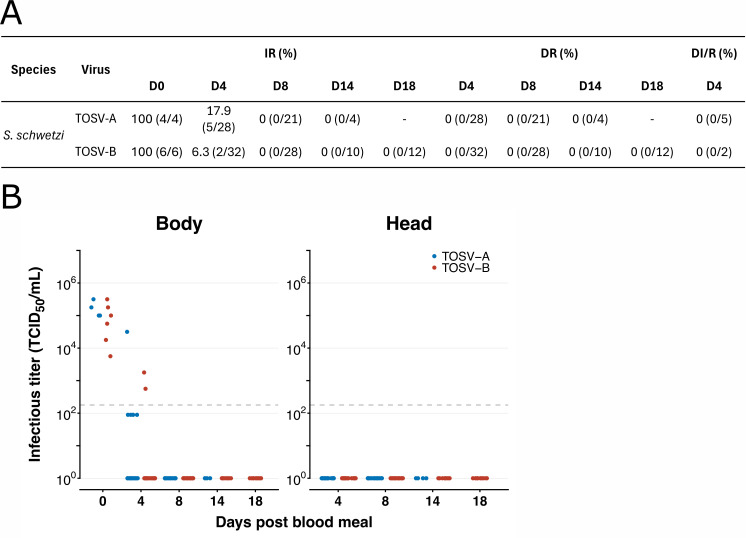
Susceptibility of *S. schwetzi* to TOSV infection. *S. schwetzi* sand flies were orally exposed to blood meal with either TOSV A or TOSV B (10^6^ PFU/ml) and collected at D4, D8, D14 and 18 p.i. to analyse their body and head with salivary glands for the presence of TOSV. (**A**) Summary values of infection rate (IR), dissemination rate (DR) and dissemination rate among infected sand flies (DI/R). (**B**) Infectious viral titers were obtained by TCID_50_ limiting-dilution assays in body and head homogenates. Each dot represents a single sand fly. Light grey dashed lines indicate the threshold of virus quantification (1.78x10^2^ TCID_50_/ml).

Five feeding experiments were performed with *S. schwetzi* and 135 (53 females for TOSV A, 82 females for TOSV B) females were dissected and tested by TCID_50_ assay (Fig 4A). Sand fly females (4 for TOSV A, 6 for TOSV B) were all positive at D0 p.i., where the mean value of viral titers was 1.74 x10^5^ and 1.12x10^5^ TCID_50_/ml for TOSV A and TOSV B, respectively. 53 sand fly females were tested after infection with TOSV A, from which only one female clearly showed a positive body, while 4 others, with visible remnants of the blood meal in the midgut, were at the detection limit of TCID_50_ assay at D4 p.i. In the case of TOSV B, 2 out of 32 females were positive at D4 p.i., although with low viral loads (1.78x10^3^ and 5.62x10^2^ TCID_50_/ml) in the body (Fig 4B). These 2 individuals had visible blood meal remnants in the midgut and no viral disseminated infection was observed. Furthermore, the 28, 10 and 12 females tested at D8, D14 and D18 p.i. respectively were all negative for TOSV by TCID_50_ assay. Detection of negative strand TOSV RNA was performed on 30 females (8 from TOSV A and 22 from TOSV B). TOSV A RNA was detected in 2 female bodies at D4 p.i., of which 1 was also positive for positive strand RNA. 4 females (3 from D4 p.i. and 1 from D14 p.i.) had bodies with detectable negative strand TOSV B RNA as well as for positive-strand RNA. However, all measured TOSV B RNA copy numbers were low (from 9.8x10^3^ to 3.4x10^3^ RNA copies/µl) and the Cp values for positive strand RNA were high (from 32.5 to 38.4). Results are summarised in S1 Table.

To summarize, TOSV TCID_50_ positive or on detection threshold of TCID_50_ samples were detected at D4 p.i., in addition to females with blood meal residues, but not at any later days p.i.. However, positive RNA TOSV strands were detected in three TCID_50_-negative females, implying replication, but no dissemination.

## Discussion

The circulation of arboviruses in nature relies on complex interrelationships among the virus, vector and vertebrate hosts, that are influenced by several factors [44]. In this study, we focused on vector competence. For arbovirus transmission by mosquitoes, the following steps are necessary: (i) infection of gut cells, (ii) virus dissemination from the midgut to secondary tissues and (iii) infection of salivary glands followed by virus release into saliva. Arboviruses thus must overcome the following barriers to be transmitted: (i) midgut infection barrier (MIB), (ii) midgut escape barrier (MEB), (iii) salivary gland infection barrier (SGIB) and (iv) salivary glands escape barrier (SGEB) [44–47]. Other important factors include genetic factors [48,49], infection dose [50–52], immunity [53–55], gut microbiome [56–58], co-infections [59,60] and temperature [61–63].

Here, we assessed the vector competence of four sand fly species: *P. tobbi*, *P. sergenti*, *P. papatasi* and *S. schwetzi* for genetically distinct TOSV strains from lineage A and B. We used two methods, TCID_50_ assay and qPCR because viral RNA level is not always directly correlated to the infectivity of virus. According to our results, the molecular detection is more sensitive compared to the TCID_50_ assay, however, the benefit of this assay is that it can detect infectious viral particles. Altogether, we observed a good correlation between viral RNA quantities and titers.

TOSV B appeared to be more successful in terms of infection and dissemination in sand flies than TOSV A at least for the strains tested. *P. tobbi* appeared be the most susceptible species. To our knowledge, only TOSV of lineage A has been detected in *P. tobbi* [31]. However, this could be due to bias in field trapping methodology, such as testing in pools based on location and date of collection without specifying the species, combined with the limited sequence data available for sand flies. In the Eastern Mediterranean area, where two main TOSV lineages circulate, *P. tobbi* is indeed present [3]. Thus, it may only be a matter of time before lineage B is detected in this species. Our experimental results and the spread of TOSV over the last decade suggest that *P. tobbi* plays a role in the circulation and transmission of this virus, warranting further research on this vector-TOSV combination. Moreover, *Phlebotomus tobbi* is the major vector of *Leishmania infantum*, *L. donovani* and their hybrids, causing visceral and cutaneous leishmaniases in the Balkans, Turkey, Cyprus and Middle East [64–66]. There are currently almost no information or experimental research on the co-infection of TOSV and *Leishmania*, as well as their potential interaction in sand flies. Future studies are needed to explore this relationship. This study paves the way for further research in this area.

In the case of *P. sergenti*, a strong midgut barrier seems to prevent the dissemination of TOSV B strain, with a significantly lower infection rate than *P. tobbi* (61% vs 7.2%). However, when the virus did overcome these barriers, it disseminated to the head. Infection conditions for *P. tobbi* and *P. sergenti* may of course be different under natural conditions. In nature, females may blood feed repeatedly during one gonotrophic cycle, increasing vector competence as described for dengue, Zika and chikungunya viruses in mosquitoes [67]. It has been shown that infectious blood meal followed by a non-infectious blood meal increases the dissemination rate compared to single-fed females, probably due to blood meal-induced microperforations in the basal lamina surrounding the midgut epithelium that increases the probability of virus escape [67]. *Phlebotomus sergenti* is a highly anthropophilic sand fly and the primary vector of *Leishmania tropica* causing cutaneous leishmaniasis in Magreb area, Middle East, Turkey and Crete [36,68]. Thus, its susceptibility to TOSV has important epidemiological consequences.

To definitively confirm these two sand fly species as new TOSV vectors according to World Health Organization (WHO) criteria [69], transmission experiments between sand flies and laboratory animal models or detection of TOSV from saliva would be necessary. However, conducting such experiments with sand flies presents significant challenges. They are considerably more fragile than mosquitoes, exhibiting lower feeding and survival rates under laboratory conditions. This was evident in our experiments, where, for example, *P. tobbi* did not survive beyond D8 p.i.. Furthermore, individuals that survived the initial blood feeding were highly reluctant to feed again, making it nearly impossible to obtain a sufficient sample size for the transmission experiments.

*Sergentomyia schwetzi* is refractory to TOSV B strain infection. Only 2 positive females at D4 p.i. were positive, likely related to blood meal residues in the midgut. None of the females tested at later time points was positive for TOSV. We tested this species since TOSV RNA has been previously detected in *S. minuta* in France [30] and *S. schwetzi* is its closest relative species, colonized in the laboratory, and willing to blood feed on an artificial system. Although *S. schwetzi* could be different than *S. minuta* in terms of TOSV vector competence, we believed that it was important to determine if this species, found in Africa, could play a role in TOSV transmission [11,33,70,71]. Our data suggest that *S. schwetzi* is not a TOSV competent vector.

Similarly, negative results were obtained for *P. papatasi* which also appears to be resistant to TOSV infection. *Phlebotomus papatasi* is widespread across Europe, Africa and Asia and is a well-known peridomestic and anthropophilic sand fly, recognized for its aggressive behaviour [36]. In recent years, its range has expanded northward into new regions, including the Balkans and Eastern Europe, up to Moldova [72,73]. Therefore, our finding that this species does not contribute to TOSV circulation has important epidemiological implications for all areas where *P. papatasi* is present.

A different situation has been observed with TOSV A. Various TOSV strains belonging to lineage A have been repeatedly detected in sand flies caught in the field, either from non-specified pools [70,74] or from pools of *S. dentata*, *P. papatasi*, *Phlebotomus* sp. [35], *P. tobbi*, *P. perfiliewi* s.l. [31], *P. perniciosus* [75] or individually from *P. major* s. l. [35]. However, in this study, the TOSV A strain tested did not appear to have much infectivity in any of the tested species; as females from D4 to D14 were completely negative. Whether this is due to the virus strain *per se*, its passage history of the virus, or genuine biological factors, remains to be determined. However, it cannot be excluded that infections detected in nature may be accidental. The development of a reverse genetics system for TOSV may be key to identify the viral determinant, if any, favouring TOSV B infection and dissemination ins and flies compared to TOSV A [76].

Overall, our results suggest that besides *P. perniciosus* and *P. perfiliewi*, other important anthropophilic sand fly species, particularly *P. tobbi* and possibly *P. sergenti,* play a role in TOSV transmission. To our knowledge, this is the first successful viral infection of these species in a laboratory setting. Nevertheless, confirmation of TOSV transmission to the host is still required for the definitive confirmation of these species as TOSV vectors.

## Supporting information

S1 Table Samples that were analyzed in this study. The first sheet contains samples of *P. tobbi*, while the subsequent sheets contain samples of *P. sergenti*, *P. papatasi*, and *S. schwetzi*, respectively. The columns in the table indicate the following: the number of blood feeding experiment of each sand fly species, the specific code of each blood feeding experiment, the specific code of the sample, and the number of the sample with an explanation of which body part of sand fly it is (H - head with salivary glands, B - the rest of the body). The subsequent columns provide: information about the strain of TOSV (either TOSV A or TOSV B) that was used for blood feeding, the result of the TCID_50_ assays, dissemination of TOSV in the sand fly (positive both head and body), the virus titers obtained by the TCID_50_ assay (TCID_50_/ml), and notes about the results of the TCID_50_ assay. The final six columns present the data from quantitative PCR (qPCR): the mean (from technical triplicate) crossing point (Cp) value of negative TOSV strand RNA measured by qPCR, standard deviation (STD) of the Cp value of TOSV negative strand RNA, the genome copy number counted from the sample Cp value of TOSV negative strand RNA and standard curve, STD of the genome copy number, the mean (from technical triplicate) Cp value of the TOSV positive strand RNA measured by qPCR and STD of the Cp value the TOSV positive strand RNA.”.
(XLSX)
